# Concurrent hyperfractionated chemoradiotherapy for head and neck squamous cell carcinoma: the prognostic impact of the overall treatment time and completion rates of chemotherapy

**DOI:** 10.1186/s40064-015-1244-8

**Published:** 2015-08-25

**Authors:** Masami Fujii, Takayuki Ohguri, Katsuya Yahara, Hajime Imada, Kyosuke Tomura, Mai Sakagami, Gunji Nagatani, Hideaki Suzuki, Yukunori Korogi

**Affiliations:** Department of Radiology, University of Occupational and Environmental Health, 1-1 Iseigaoka, Yahatanishi-ku, Kitakyushu, 807-8555 Japan; Department of Cancer Therapy Center, Tobata Kyoritsu Hospital, Kitakyushu, Japan; Department of Otorhinolaryngology-Head and Neck Surgery, University of Occupational and Environmental Health, Kitakyushu, Japan

**Keywords:** Concurrent chemoradiotherapy, Head and neck cancer, Hyperfractionation, Carboplatin

## Abstract

The purpose of this study was to investigate whether the overall treatment time and completion rates of chemotherapy were predictive factors for the survival rates in patients with squamous cell carcinoma of the head and neck (SCCHN) who were treated with concurrent chemoradiotherapy (CCRT) using hyperfractionated radiotherapy (RT) and daily carboplatin. The number of intermission days of RT were as follows; 0 (n = 37), 1–5 (n = 8), 6–10 (n = 12) and ≥11 (n = 12), and the days of RT without carboplatin; 0 (n = 27), 1–5 (n = 13), 6–10 (n = 13) and ≥7 (n = 16). The overall treatment time (≤48 vs ≥49 days) was a significant prognostic factor for the local control, disease-free survival and overall survival rates. The completion rate of chemotherapy, as the number of days of RT without carboplatin, was not a significant factor affecting any of the survival rates. In discussion, the strengths of the present study contain that all the patients were treated with 72 Gy delivered as 1.2 Gy twice daily, and received concurrent chemotherapy comprising daily carboplatin as a radio-sensitizer. Based on the results, the completion rate of chemotherapy may have a lower impact on the local control rate in comparison with the overall treatment time. We believe that when a treatment interruption is needed because of the acute toxicities, hyperfractionated RT should be resumed as soon as possible independently while continuing the break of daily carboplatin. The overall treatment time influenced the clinical outcomes in SCCHN patients treated with hyperfractionated CCRT using carboplatin, while the impact of the completion rates of daily carboplatin was limited. Sixty-nine consecutive patients with SCCHN were initially treated with definitive CCRT and were retrospectively analyzed. All 69 patients were treated with CCRT using hyperfractionated RT of 72 Gy in 60 fractions and daily carboplatin (25 mg/m^2^). The patients treated with other chemotherapeutic regimens or induction chemotherapy were excluded. On the intermission days of the RT, carboplatin was not prescribed. After the intermission, CCRT using RT plus daily carboplatin or RT alone was resumed.

## Background

The prolongation of the overall treatment time of radiotherapy (RT) results in poorer clinical outcomes in patients with squamous cell carcinoma of the head and neck (SCCHN) (Duncan et al. 1996). Hyperfractionated RT has been used for head and neck cancers, especially for advanced cases, because it reduces the opportunity for the proliferation of tumor cells and increases the efficiency of tumor control without increasing late toxicity. Phase III randomized trials have demonstrated a significant improvement in the local control of head and neck cancers treated with twice-daily fractionation over once daily fractions (Horiot et al. 1992). Chemoradiotherapy has also been studied intensively in patients with SCCHN, (Marcial et al. 1990; Munro 1995). Concurrent chemoradiotherapy (CCRT) is attractive because chemotherapeutic agents may act as radiosensitizers, and the overall treatment time is not prolonged (Munro 1995). In the meta-analyses of head and neck cancers, the survival benefit of chemotherapy given synchronously with RT has been demonstrated (Munro 1995). In this context, concurrent chemoradiotherapy (CCRT) and hyperfractionated RT have been used for SCCHN, and the efficacy of CCRT using hyperfractionated RT was confirmed in a meta-analysis (Budach et al. 2006).

There have been only a few reports that have investigated whether the overall treatment time or completion rate of concurrent chemotherapy is a prognostic factor in SCCHN patients treated with CCRT using conventional fractionated RT (Pignon et al. 2000; Langendijk et al. 2004). However, to the best of our knowledge, there have been no reports that have evaluated the prognostic impact of the overall treatment time or completion rate of concurrent chemotherapy in patients treated with CCRT using hyperfractionated RT. The purpose of this study was therefore to investigate the prognostic impact of the overall treatment time and completion rates of chemotherapy in patients with SCCHN who were treated with CCRT using hyperfractionated RT.

## Results and discussion

The follow-up ranged from 3 to 120 months (median, 46 months). Table 1 shows the number of intermission days of RT and days of RT without carboplatin. The 3-year LC, DFS, DMFS and OS rates in all 69 patients were 66, 62, 90 and 75 % respectively. Local recurrence and distant metastasis developed in 24 (39 %) and seven (10 %) patients, respectively.

**Table 1:** The intermission of RT and days of RT without carboplatin

Days	Intermission days of RT n (%)	Days of RT without carboplatin n (%)
0	37 (54)	27 (39)
1–5	8 (12)	13 (19)
6–10	12 (7)	13 (19)
11–20	11 (16)	13 (19)
21–	1 (1)	3 (4)

**Fig. 1:**
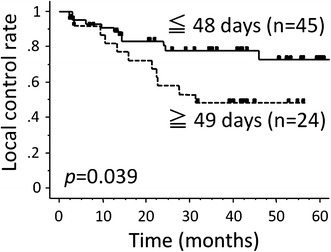
The overall treatment period (≤48 days) was a significant predictor of the local control rate (p = 0.039)

**Fig. 2:**
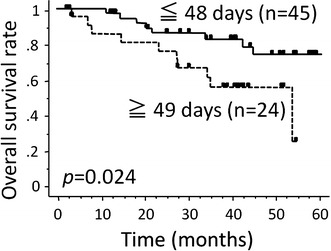
The overall treatment period (≤48 days) was a significant predictor of the overall survival rate (p = 0.024)

**Fig. 3:**
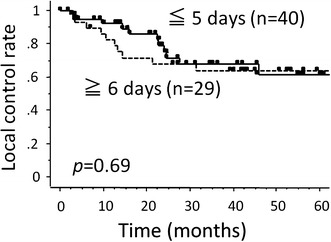
The rate of completion of chemotherapy, as the number of days of RT without carboplatin, was not a significant predictor of the local control rate

Table 2 summarizes the results of the univariate analyses by the Kaplan–Meier approach with log-rank testing to evaluate the impact of certain factors on the survival rates. The number of overall treatment days was a significant prognostic factor for the LC, DFS and OS rates (Figs. 1, 2). The completion rate of chemotherapy, as the number of days of RT without carboplatin, was not a significant factor associated with any of the survival rates (Fig. 3). The clinical stage was a significant factor associated with the LC, DFS DMFS and OS. The T stage was also a significant predictor of the LC, DFS and OS.

**Table 2:** The results of the univariate analyses of factors predicting the survival rates

	n	Local control		Disease-free survival		Distant metastasis-free survival		Overall survival	
		3-year (%)	*p*	3-year (%)	*p*	3-year (%)	*p*	3-year (%)	*p*
Age (years)			0.41		0.44		0.81		0.82
<65	33	72		67		87		79	
≥65	36	62		59		91		70	
PS			0.73		0.63		0.83		0.34
0–1	59	68		64		87		77	
2–3	10	64		58		100		61	
Clinical stage			0.007		0.0009		0.01		<0.0001
I–II	35	84		84				100	
III–IV	34	50		39				49	
Tumor stage			<0.0001		<0.0001		0.17		<0.0001
T1–2	55	79		74		80		87	
T3–4	14	23		17		90		34	
N stage			0.05		0.0091		0.01		0.0012
N0	38	77		77		97		94	
N1–3	31	54		43		76		52	
Overall treatment (days)			0.039		0.015		0.11		0.02
≤48	45	78		74		91		84	
≥49	24	48		41		85		56	
Days of RT without carboplatin			0.69		0.61		0.82		0.21
≤5	40	68		64		83		80	
≥6	29	64		41		95		67	
Hemoglobin at the start of RT (g/dl)			0.30		0.22		0.66		0.02
<12	18	51		44		83		57	
≥12	51	71		68		92		82

In discussion, the present study is the first study to evaluate the overall treatment time of RT and the completion rates of chemotherapy for SCCHN patients treated with CCRT using hyperfractionated RT. The strengths of the present study include that all the patients were initially treated with 72 Gy delivered as 1.2 Gy twice daily, and received concurrent chemotherapy comprising daily carboplatin as a radio-sensitizer for the hyperfractionated RT (Chitapanarux et al. 2007). In addition, the patients who received neoadjuvant chemotherapy, followed by radiotherapy, were not included. Therefore, the effects of the overall treatment time and completion rates of daily carboplatin on the clinical outcomes could be evaluated with less bias. Our results confirmed that the overall treatment time was a significant factor predicting the LC, DFS and OS rates, while the completion rate of daily carboplatin was not.

In many of the previous studies of the clinical outcomes of RT alone, an interruption of RT was found to be a significant predictor of the local control (Bese et al. 2007). RT alone with a hyperfractionated schedule also showed a significant improvement in the LC and DFS in patients treated with shorter overall treatment times (Leborgne et al. 2001). However, as mentioned in the Introduction, there have been limited clinical reports about treatment interruption in SCCHN patients treated with CCRT (McCloskey et al. 2009; Gupta et al. 2009). McCloskey et al. reported that RT treatment interruption greater than 1 week was a significant predictor of worse LC after definitive CCRT using cisplatin (most common regimen 100 mg/m^2^ on days 1, 22 and 43) and conventional fractionated RT (McCloskey et al. 2009). Gupta et al. demonstrated that the total cycles (≥6 cycles) of concurrent weekly cisplatin (30 mg/m^2^), as well as a shorter overall treatment time, was a significant prognostic factors for a better LC and DFS (Gupta et al. 2009). In the current study of CCRT using hyperfractionated RT and daily carboplatin, although the rate of completion of chemotherapy was not a prognostic factor, the overall treatment time was a significant predictor. We supposed that the completion rate of chemotherapy may have a lower impact on the LC rate in comparison with the overall treatment time, because the daily low-dose regimen was prescribed mainly as a radio-sensitizer, and the antitumor effects of hyperfractionated RT may strongly depend on the overall treatment time (Leborgne et al. 2001).

A prolonged overall treatment time is a well-known factor that has a negative impact on the outcome of SCCHN due to tumor cell repopulation (Hoffstetter et al. 1997; Tarnawski et al. 2002; Suwinski et al. 2003). Nishimura et al. reported that an overall treatment time longer than 49 days was significantly associated with a poor local control rate in patients with T1 or T2 glottic carcinoma; a 1-week interruption of RT significantly reduced the 5-year local control probability of T1 glottic tumors from 89 to 74 % (Nishimura et al. 1996). Kawashima et al. also indicated that the patients who had received 70 Gy/≤49 days for accelerated fractionation RT achieved a better local control rate than those who had received other, more conservative total dose/overall treatment time with statistical significance in patients with T2 or worse hypopharyngeal cancer (Kawashima et al. 2007). According to these clinical results, we chose ≤49 and ≥49 days as the difference in treatment time, and recommended that patients receive radiation therapy for a maximum of 48 days and no longer, because we confirmed that the overall treatment time (≤49 days) was a significant prognostic factor for the survival rates in the current study.

Hyperfractionated RT has resulted in increased severe acute complications requiring intensive nutritional support for the completion of treatment in some patients. Wang et al. suggested that a prolongation of the treatment gap for more than 14 days adversely affected the local control in patients with locally advanced head and neck cancer treated with accelerated hyperfractionated radiation therapy, and the midcourse treatment gap should be kept as short as possible (Wang et al. 1996). In a Phase III trial of hyperfractionated RT with or without concurrent chemotherapy for locally advanced head and neck cancer, combined treatment for advanced head and neck cancer was more efficacious than hyperfractionated RT alone (Brizel et al. 1998). This trial included planned treatment breaks to avoid the withdrawal of the recruited subjects due to acute toxicity; eventually, the treatment time of RT in the hyperfractionated RT arm averaged 42 days, while that in the hyperfractionated RT plus concurrent chemotherapy arm averaged 47 days (Brizel et al. 1998). However, the long-term follow-up in the Phase III Radiation Therapy Oncology Group (RTOG) 0129 trial indicated no difference in the overall survival or late toxicity with the use of accelerated-hyperfractionated vs standard RT plus cisplatin in patients with locally advanced head and neck cancer (Nguyen-Tan et al. 2014). Based on the results of the present study of CCRT using hyperfractionated RT and daily carboplatin, we believe that when a treatment interruption is needed because of the acute toxicities, hyperfractionated RT should be resumed as soon as possible independently while continuing the break of daily carboplatin.

Regarding the limitations associated with this study, it was a retrospective case series, so the possibility that there was some selection bias with regard to the prognostic factors could not be ruled out, although all of the patients received definitive CCRT using hyperfractionated RT of 70 Gy in 60 fractions and daily carboplatin, and were initially treated with non-surgical management. The treatment regimen of CCRT using hyperfractionated RT and daily carboplatin differed from the standard therapy, such as CCRT using conventional fractionation and cisplatin. However, a Phase III randomized trial of CRT using cisplatin vs. carboplatin for head and neck cancer demonstrated that there was no difference in the survival rates between cisplatin and carboplatin, and the completion rates of carboplatin was higher than that of cisplatin (Chitapanarux et al. 2007). In addition, a significant improvement in the local control of head and neck cancers treated with twice-daily fractionation over once daily fractions in Phase III randomized trials for RT monotherapy for head and neck cancer was observed (Horiot et al. 1992). Therefore, we chose this regimen presuming an improvement in the treatment results in patients with SCCHN. A formal prospective trial is necessary to determine the prognostic impact of the overall treatment time and completion rate of chemotherapy in patients treated with CCRT using hyperfractionated RT.

## Conclusions

In conclusion, this study demonstrated that the longer overall treatment time may have a negative impact on the clinical outcomes in SCCHN patients treated with concurrent CCRT using hyperfractionated RT and daily carboplatin, while the completion rates of daily carboplatin may not affect the clinical outcomes. When the acute toxicities that need the treatment interruption occur, it is important to consider the early resume of hyperfractionated RT without restart of the daily carboplatin.

## Methods

From June 1999 to January 2010, 608 consecutive patients with primary head and neck cancer were prospectively recorded in the database for RT at the authors’ institution. The following requirements had to be met for patients to be included in this retrospective study: a pathologically confirmed SCCHN, treated with concurrent chemoradiotherapy (CCRT) using hyperfractionated RT of 72 Gy in 60 fractions and daily carboplatin (25 mg/m^2^), without distant metastatic disease. The following patients were excluded: those who had been treated with neoadjuvant chemotherapy, had received transoral debulking microsurgery and those who had postoperative disease. Sixty-nine patients were selected from the database and were retrospectively analyzed. Written informed consent for treatment was obtained from all patients. The study was approved by the Institutional Review Board of the authors’ institution.

The characteristics of the patients are listed in Table 3. The performance status and tumor invasion were evaluated at the start of our treatment. Staging was achieved by clinical examination, CT and endoscopic examination, and in some cases, magnetic resonance imaging (MRI) and 18-Fluoro-deoxyglucose positron emission tomography (FDG-PET). All 69 patients were treated with two daily fractions of radiation of 1.2 Gy (total dose, 72 Gy), with a 6-h interval between fractions. Computed tomography-assisted three-dimensional treatment planning (Xio or FOCUS; CMS Japan, Tokyo, Japan) was used to determine the radiation fields. Uninvolved nodal areas of the neck were treated with 43.2 Gy/36 Fr. The primary tumor and gross nodal disease were treated with 72 Gy/60 Fr. Prophylactic neck irradiation for uninvolved nodal areas was not performed in 14 patients with T2N0M0 glottic cancer of the larynx.

**Table 3:** The patient characteristics

Variable	n = 69 (%)
Age (years)	
Median (range)	69 (40–85)
Gender	
Male	60 (87)
Female	9 (13)
Performance status	
0	12 (17)
1	46 (67)
2	10 (15)
3	1 (1)
Tumor type, TNM^a^	
Nasopharyngeal ca.	5 (7)
T1N2M0	1
T1N3M0	1
T2N1M0	3
Oropharyngeal ca.	15 (22)
T1N0M0	1
T1N2M0	4
T2N0M0	1
T2N1M0	2
T2N2M0	1
T3N1M0	1
T4N1M0	1
T4N2M0	1
T4N3M0	3
Hypopharyngeal ca.	22 (32)
T1N0M0	2
T1N2M0	1
T2N0M0	8
T2N1M0	1
T2N2M0	4
T3N2M0	2
T4N0M0	2
T4N2M0	2
Laryngeal ca.	27 (39)
T2N0M0	22
T2N1M0	1
T2N2M0	2
T3N0M0	1
T3N2M0	1

^a^International Union Against Cancer tumor, node, metastasis classification, 6th edition

**Fig. 4:**
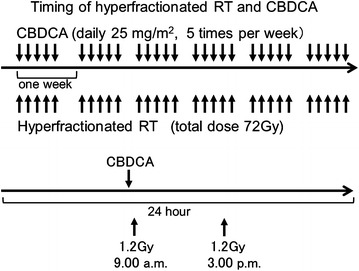
The schedules of concurrent chemoradiotherapy using hyperfractionated radiotherapy and carboplatin

The detailed irradiation and concurrent chemotherapy schedules are shown in Fig. 4. Carboplatin was planned to be administered as an intravenous bolus immediately before the first daily fraction at a dose of 25 mg/m^2^ on every treatment day. On the intermission days of the hyperfractionated RT, carboplatin was not prescribed. After the intermission, CCRT using hyperfractionated RT plus daily carboplatin or hyperfractionated RT alone was resumed.

In all patients, complete head and neck examinations, including flexible laryngoscopy, were performed after the completion of CCRT and every 1–3 months for the first year, every 2–3 months for the second and third years and every 2–6 months for the fourth and fifth years after the CCRT. CT scans were performed every 3–6 months at the discretion of the clinician, and in some patients, fluorodeoxyglucose positron emission tomography/computed tomography (FDG-PET/CT) or magnetic resonance imaging (MRI) was additionally used.

The local control (LC), defined as failure to have a recurrence within the radiation field, the disease-free survival (DFS), distant metastasis-free survival (DMFS) and overall survival (OS) rates were calculated from the start of RT using the Kaplan–Meier method. The statistical significance of the differences between the actuarial curves was assessed using the log-rank test. To identify prognostic factors for the survival rates, univariate analyses were performed using the age, performance status, clinical stage, T stage, N stage, overall treatment days, days of RT without carboplatin and the hemoglobin level at the start of RT. Multivariate analysis was not performed because of the small number of patients.
